# Evaluating Large Reasoning Models Versus Human Multidisciplinary Teams in Lung Cancer Decision-Making: Real-World Study

**DOI:** 10.2196/91733

**Published:** 2026-07-16

**Authors:** Ivan Viculin, Josip Vrdoljak, Krešimir Tomić, Ivana Canjko, Dora Čerina Pavlinović, Mateo Ćurin, Lidija Bošković, Zvonimir Družianić, Bartul Vuković, Niko Dunkic, Vide Popović, Suzana Mladinov, Joško Božić, Eduard Vrdoljak

**Affiliations:** 1 Department for Pulmonary Disease, University Hospital of Split, Split, Split-Dalmatia, Croatia; 2 Laboratory for AI in Biomedicine, School of Medicine, University of Split, Split, Split-Dalmatia, Croatia; 3 Department of Oncology, University Clinical Hospital Mostar, Mostar, Federation of B&H, Bosnia and Herzegovina; 4 Department of Oncology, University Hospital Centre Osijek, Osijek, County of Osijek-Baranja, Croatia; 5 Department of Oncology, University Hospital of Split, Spinčićeva 1, Split, Split-Dalmatia, Croatia; 6 Laboratory for AI in Biomedicine, Department of Pathophysiology, School of Medicine, University of Split, Split, Split-Dalmatia, Croatia; 7 Department of Oncology, University Hospital of Split, Spinčićeva 1, Split, Split-Dalmatia, Croatia, 385 98448431

**Keywords:** large reasoning models, large language models, clinical decision-making, lung cancer, artificial intelligence

## Abstract

**Background:**

Large language models (LLMs) and large reasoning models (LRMs) have shown excellent performance on medical benchmarks, although evaluations concerning real-world medical workflows are still lacking. Lung cancer care is particularly dependent on the multidisciplinary team (MDT) integration of radiology, pathology, staging, and treatment planning, making it a high-bar setting for evaluating LRMs.

**Objective:**

This study aimed to compare the quality of recommendations generated by 2 LRMs (GPT-5-Thinking and Deepseek-v3-r1) with each other and with human MDT decisions in real-world lung cancer cases, as well as to assess whether MDT awareness of AI comparison influences the quality of MDT decisions.

**Methods:**

This was a single-center real-world comparative study of 100 consecutive lung cancer MDT cases (50 retrograde and 50 anterograde) from the University Hospital of Split, Croatia. For each case, deidentified structured reports (containing all necessary patient or case data, while excluding MDT conclusions) were submitted once to GPT-5-Thinking and Deepseek-v3-r1 to generate recommendations for radiologic diagnostics, pathologic diagnostics, oncologic therapy, and overall usefulness. Two independent lung oncologists graded MDT decisions and model outputs on 1-5 Likert scales. An average recommendation score (avg_rec) was calculated as the mean of radiology, pathology, and therapy scores. Analyses used Wilcoxon tests for paired model comparisons, Mann-Whitney tests for between-phase comparisons, and Spearman correlations (2-sided α=.05).

**Results:**

Ratings showed ceiling effects. In the retrograde phase (N=50), the mean (95% CI) GPT-5-Thinking scores were higher than Deepseek-v3-r1 scores for radiologic diagnostics (4.89, 4.78-4.99 vs 4.76, 4.62-4.89; *P*<.001), oncologic therapy (4.82, 4.69-4.94 vs 4.18, 3.82-4.54; *P*<.001), and usefulness (4.82, 4.69-4.94 vs 4.18, 3.84-4.53; *P*<.001); pathologic diagnostics were similar (4.88, 4.78-4.97 vs 4.73, 4.57-4.90; *P*=.15). In the anterograde phase (n=50), the mean (95% CI) GPT-5-Thinking scores remained higher for radiology (4.94, 4.85-5.03 vs 4.64, 4.47-4.81; *P*<.001) and pathology (4.96, 4.90-5.02 vs 4.78, 4.65-4.91; *P*=.008), with smaller differences for therapy (4.46, 4.18-4.74 vs 4.20, 3.86-4.54; *P*=.24) and usefulness (4.50, 4.24-4.76 vs 4.16, 3.83-4.49; *P*=.12). The mean (95% CI) GPT-5-Thinking avg_rec exceeded MDT grade in both phases (retrograde: 4.90, 4.84-4.95 vs 4.14, 3.96-4.33; *P*<.001; anterograde: 4.79, 4.69-4.89 vs 4.34, 4.16-4.52; *P*<.001); Deepseek-v3-r1 exceeded MDT in the retrograde phase (4.56, 4.40-4.72 vs 4.14, 3.96-4.33; *P*<.001) but not the anterograde phase (4.54, 4.41-4.67 vs 4.34, 4.16-4.52; *P*=.15). MDT grades did not differ between phases (*P*=.13).

**Conclusions:**

In 100 real-world lung cancer MDT cases, both LRMs produced high-quality recommendations, with GPT-5-Thinking consistently outperforming Deepseek-v3-r1 and exceeding expert-graded MDT decision quality in both phases. MDT decision quality was unchanged by awareness of AI benchmarking. LRMs can thus generate recommendations comparable to or exceeding expert MDT decisions, though the single-center design and ceiling effects limit generalizability. Whether integrating such tools into MDT workflows improves clinical decisions warrants prospective study.

## Introduction

Recent advances in artificial intelligence, particularly large language models (LLMs), have generated significant interest in their potential for clinical decision support. The advent of models like ChatGPT has sparked widespread investigations into the ability of AI to augment health care practice [1]. Early explorations suggest these LLMs can yield promising results across diverse medical tasks [1]. By processing large amounts of unstructured clinical data, such models exhibit a strong capacity to interpret complex medical information [2]. This has led to the notion of large reasoning models (LRMs), denoting advanced AI systems engineered for sophisticated problem-solving (with long chains of thought and greater token consumption per problem), which are being actively explored as tools to assist clinicians in decision-making [3].

In oncology, recent studies have shown both the promise and limitations of state-of-the-art LLMs and LRMs. For example, an open-source LLM, DeepSeek-v3-r1, demonstrated high accuracy in retrospective lung cancer case analyses, significantly outperforming junior oncologists on diagnostic and treatment decision questions [4]. Likewise, GPT-4 (OpenAI) with vision capabilities achieved lung cancer diagnostic accuracy approaching that of experienced physicians [5]. These findings illustrate the potential for AI to match expert knowledge in specialized domains. However, they also highlight important caveats. The AI models sometimes committed reasoning errors or displayed gaps in judgment; for instance, DeepSeek’s answers, while often correct, showed a higher rate of ethically concerning reasoning compared to humans [4]. In head-to-head evaluations, even the best models did not perform perfectly: accuracy tops out around 80% to 90% on controlled question benchmarks [6]. DeepSeek achieved an 86.9% accuracy rate on oncology queries (higher than other chatbots like GPT-4), yet none of the models reached 100% [6]. Accordingly, investigators have emphasized that current LLMs or LRMs “have not yet achieved sufficient accuracy for standalone clinical use” and require rigorous validation before real-world deployment [6]. In summary, today’s most advanced models (eg, Deepseek-v3-r1) show near-expert performance in oncology tasks, but they remain imperfect and unproven in live clinical settings [4,6]. On the other hand, the quality of work of MDT itself is often questionable. Not all institutions have properly staffed MDTs, and not all MDT decisions are according to the guidelines or in the best interest of individual patients, optimally personalized. Consequently, the most probable future lies in the optimal integration of both worlds: human and AI.

A critical limitation of prior research is the context in which LLMs have been evaluated. Most studies to date rely on simulated or retrospective data rather than in real-world clinical decision-making cases. Common approaches include testing AI models on curated case vignettes, question-and-answer sets derived from guidelines, or curated historical patient datasets [7]. Such benchmarks are informative, but they do not fully capture the complexity and immediacy of real multidisciplinary team discussions. Notably, very few studies have directly compared AI recommendations to the decisions of human clinicians in an anterograde way. Even the consistency and quality of human multidisciplinary team (MDT) decisions have rarely been formally assessed in the literature [8], underscoring a broader gap in understanding decision-making dynamics. This lack of real-world comparative studies means it remains largely unknown how an LLM or LRM would perform when faced with the authentic, multifaceted scenarios that expert teams navigate in practice.

Clinical decision-making in oncology (especially in lung cancer) is a complex, high-stakes process that typically demands MDT consensus. Lung cancer management exemplifies why expert deliberation is critical: optimal care often requires integrating input from pulmonologists, radiologists, thoracic surgeons, medical and radiation oncologists, pathologists, and other specialists. Accordingly, MDT case conferences have become the standard of care worldwide for planning diagnostics and treatment in cancer [8]. These team discussions ensure the accurate staging of the disease and the selection of the best possible treatment plan for each patient. This intricate, collaborative decision process sets a high bar for any AI system aspiring to emulate or assist an MDT in practice.

Given the current progress of AI and the limited evidence from real clinical workflows, a direct comparison of large reasoning models with human experts is both timely and necessary. This study addresses that gap by evaluating 2 advanced models (GPT-5-Thinking and Deepseek-v3-r1) against the decisions of a MDT for lung cancer, using real-world data from 100 consecutive cases at a national oncology center. We compared model-generated diagnostic and therapeutic recommendations with the MDT’s consensus decisions to assess whether state-of-the-art AI can approach the quality of multidisciplinary decision-making in complex oncology care.

A secondary aim was to determine whether the behavior of the MDT changes under evaluation. In a retrograde phase, we analyzed historical MDT decisions made without AI, whereas in an anterograde phase, the MDT was aware that its decisions would be compared to model outputs. This design allowed us to examine whether awareness of an AI “observer” influences decision-making, and to inform the safe and effective integration of large reasoning models into multidisciplinary cancer care.

## Methods

### Study Design and Data Source

This comparative real-world study evaluated the performance of LRMs against an MDT of human experts in lung cancer decision-making. The study was conducted at the Clinic for Oncology, University Hospital of Split, Croatia, using routinely collected MDT documentation. All consecutive lung cancer cases discussed at the thoracic oncology MDT during the study period were eligible if the clinical documentation was complete and the final MDT decision was available; no case complexity or subtype selection criteria were applied. Specifically, cases were required to have an anonymized MDT summary containing tumor characteristics, radiologic and pathologic findings, disease stage, comorbidities where relevant, and a documented MDT recommendation regarding further diagnostics and oncologic treatment. In accordance with General Data Protection Regulation requirements, exact patient ages were removed during deidentification; only age brackets were retained and provided to the models. Patient ages ranged from 45 to 87 years. The thoracic oncology MDT comprised the following core members: a pulmonologist with 11 years of clinical experience, a senior medical oncologist with 37 years of clinical experience, a medical oncologist with 7 years of clinical experience, a thoracic surgeon with 10 years of clinical experience, a diagnostic radiologist specializing in thoracic imaging, and a pathologist with expertise in pulmonary and thoracic pathology. All MDT members held attending-level (specialist) appointments at the University Hospital of Split. Additional specialists (eg, a radiation oncologist and a nuclear medicine physician) were consulted on a case-by-case basis as clinically indicated.

Two cohorts of cases were assembled. The retrograde cohort comprised cases of lung cancer cases discussed before the MDT members were informed about the study and the planned comparison with LRMs. The anterograde cohort comprised lung cancer cases discussed after MDT members had been informed that their decisions would later be compared with AI-generated recommendations. In both cohorts, the MDT functioned as usual and made real-time clinical decisions. An a priori sample size calculation was performed for the primary paired analysis (Wilcoxon signed-rank test comparing LRM and MDT scores within each case). Assuming a 2-sided α of .05 and 80% power, and adopting a conservative small-to-medium effect size (Cohen *d*=0.30) given the absence of prior LRM-vs-MDT benchmarks in oncology, the calculation yielded a minimum requirement of 92 paired observations. We therefore set the target at 100 cases (50 per study phase), providing a modest safety margin and a convenient round number. For this analysis, 50 retrograde and 50 anterograde MDT cases were sampled, yielding a total of 100 unique patient cases. All patient identifiers were removed before LRM prompting and grading; only deidentified patient reports were used.

### LRMs and Report Generation

Two state-of-the-art LRMs were evaluated: GPT-5-Thinking (OpenAI) and Deepseek-v3-r1 (Deepseek). These 2 models were selected because, at the time of data collection, they represented the highest-performing proprietary (closed-source) and open-weight (open-source) large reasoning models, respectively, on established general-purpose and reasoning benchmarks. The selection was therefore based on overall reasoning capability rather than domain-specific oncology benchmarks, which do not exist for LRMs in a standardized form. Including one proprietary and one open-weight model also allowed for a comparison across deployment paradigms (API-only vs potentially self-hosted). Both models were accessed via their respective official APIs (OpenAI API for GPT-5-Thinking; Deepseek API for Deepseek-v3-r1) using default inference parameters (temperature=1.0; top_*P*=1.0) and treated as black boxes; no model fine-tuning was performed. For each of the 100 anonymized MDT cases, the deidentified patient report was appended to a standardized prompt instruction and submitted to each model. Critically, the patient reports used as input were not curated case vignettes but rather the actual clinical documentation prepared for the MDT meeting, with only patient identifiers removed. These reports retained the unstructured, heterogeneous character of real-world clinical records: they typically included a free-text clinical anamnesis (presenting complaint, medical history, comorbidities, current medications, and performance status), verbatim radiology reports (computed tomography, positron emission tomography, magnetic resonance imaging, and bone scintigraphy), verbatim pathology and cytology reports (including immunohistochemistry and molecular testing results), laboratory values in varying formats, and procedural notes (eg, bronchoscopy and biopsy). Importantly, all inputs were text-based: radiological and pathological findings were provided as the written reports generated by the respective specialists, not as raw imaging data (eg, computed tomography scans, histopathological slides, or digitized images). This text-only input ensured a fair comparison between the 2 models, as both GPT-5-Thinking and Deepseek-v3-r1 received identical information in the same modality, and no multimodal (image-processing) capabilities were involved. The reports varied substantially in length, completeness, and internal organization across cases, reflecting real clinical practice rather than a standardized template. In some cases, information was incomplete or findings from different dates were presented in nonchronological order. The MDT conclusion was excluded from the data provided to the model. A representative anonymized example of a case report is provided in Multimedia Appendix 1, and the corresponding GPT-5-Thinking model output for that case is provided in Multimedia Appendix 1. The prompt then requested that the model provide three sets of structured recommendations: (1) recommendations for further radiologic diagnostics, (2) recommendations for further pathologic diagnostics, and (3) recommendations for oncological therapy (systemic and local treatment, including radiotherapy, surgery, and palliative options as appropriate).

The prompt used several established prompt engineering techniques: persona prompting (assigning the model the role of a board-certified oncologist), structured output specification (requiring standardized sections for case summary, diagnostics, therapeutics, and discussion points), and explicit evidence-grounding instructions (directing citation of National Comprehensive Cancer Network and European Society for Medical Oncology guidelines). A zero-shot, single-turn design was used, meaning each case was submitted once without iterative refinement or few-shot examples, reflecting a realistic clinical deployment scenario. The same prompt template was used for both models and all cases without model-specific optimization; each model was prompted once per case to avoid post hoc selection of more favorable outputs. The full prompt is provided in Multimedia Appendix 1. Model responses were exported in text form, lightly formatted for readability without altering clinical content, and stored for subsequent expert grading alongside the corresponding human MDT report for that case. Both models were accessed via their respective commercial APIs. GPT-5-Thinking was priced at US $0.625 per million input tokens and US $5.00 per million output tokens; Deepseek-v3-r1 was priced at US $0.55 per million input tokens and US $2.19 per million output tokens. The average total input per case was approximately 1800 tokens (730-token prompt template plus an average 1074-token patient report). GPT-5-Thinking generated an average of approximately 1400 output tokens per case, while Deepseek-v3-r1 generated approximately 2100 output tokens per case, resulting in estimated per case inference costs of approximately US $0.008 for GPT-5-Thinking and US $0.006 for Deepseek-v3-r1 (total cost for the full 100-case study: less than US $1 per model). Deepseek-v3-r1 is an open-weight model (671 billion parameters) that can alternatively be self-hosted; full-precision deployment requires approximately 1.3 TB of graphic processing unit memory (eg, a cluster of 8 NVIDIA A100 80 GB or equivalent), though quantized variants can run on more modest hardware. GPT-5-Thinking is a proprietary model available only via API.

### Expert Graders and Outcome Measures

Two board-certified lung oncologists, with 7 and 10 years of postresidency experience, respectively, served as expert graders. To reduce potential grading bias, the expert reviewers were drawn from independent medical centers, namely the University Hospital of Mostar and the University Hospital of Osijek. They were familiar with national and international lung cancer guidelines and with the local MDT workflow. Graders were blinded to the identity of the model that produced each output; for each case, the MDT decision and the 2 model outputs were presented without labels indicating their source. Each grader independently scored all outputs before any cross-comparison. Following independent grading, the 2 graders reviewed discrepant scores and reached a consensus grade through discussion; it is these consensus-adjudicated values that are reported in the primary analysis. For each case, the graders evaluated both the real-world MDT decision and the outputs of GPT-5-Thinking and Deepseek-v3-r1 using the same set of rating scales.

All evaluations were performed on 1‐5 Likert scales, where higher scores indicated better performance. Four domains were assessed for each model output: (1) recommendation for further radiologic diagnostics; (2) recommendation for further pathologic diagnostics; (3) recommendation for oncological therapy; and (4) overall usefulness or clinical relevance of the model’s output for real-world lung cancer decision-making, which served as a holistic assessment integrating reasoning quality, completeness, guideline concordance, safety of recommendations, and actionability for the treating team. In addition, for each case, the graders assigned an “Overall Grade of MDT” on a 1‐5 scale reflecting the perceived quality, completeness, and guideline-concordance of the human MDT decision. The grading rubric for all scales was discussed in advance by the 2 oncologists to ensure a shared understanding of what constituted optimal, mostly correct, partially correct, and inadequate recommendations.

For statistical analysis, a single value per case, model, and domain was available in the dataset. These values correspond to the final adjudicated grades used in routine evaluation and represent the outcome of the grading process for that case. From the three 1‐5 recommendation domains (radiologic, pathologic, and therapy), an aggregate “average recommendation score” (avg_rec) was calculated for each model and case as the arithmetic mean of the 3 domain scores, yielding a summary measure of overall recommendation quality on the same 1‐5 scale.

### Retrograde Versus Anterograde Comparison

The primary objective of the study was to compare the quality of GPT-5-Thinking and Deepseek-v3-r1 recommendations against each other and against expert-graded human MDT decisions in real-world lung cancer cases. A secondary objective was to explore whether making MDT members aware of the planned AI comparison influenced either MDT quality or the relative performance of the LRMs. To this end, analyses were stratified by phase. The retrograde phase reflected historical MDT decisions made under usual practice, without any awareness of subsequent AI benchmarking. The anterograde phase reflected prospective MDT decisions made after the MDT members had been informed that their outputs would later be compared with LRM recommendations.

For each phase, paired comparisons between GPT-5-Thinking and Deepseek-v3-r1 were conducted at the case level for the 3 recommendation domains and the usefulness score. In addition, phase-stratified analyses examined how model performance related to the quality. MDT grades were treated as an ordinal indicator of MDT quality and were used both as a continuous variable and in a dichotomized form, with grades 4 or less versus 4 defining lower- and higher-quality MDT decisions, respectively.

### Statistical Analysis

All analyses were performed at the case level. The sample size of 100 paired cases (50 per phase) was determined a priori to provide 80% power to detect a small-to-medium effect size (Cohen *d*=0.30) at a 2-sided α of .05 using the Wilcoxon signed-rank test, as detailed in the “Study Design” section. Because all outcomes were based on Likert-type scales with evident ceiling effects, we prespecified the use of nonparametric tests and did not assume normality of the underlying distributions. Descriptive statistics are reported as means and 95% CIs, as well as medians with IQR, and counts with percentages where appropriate.

Within each phase, paired comparisons of GPT-5-Thinking and Deepseek-v3-r1 were carried out using Wilcoxon signed-rank tests for each of the 4 model-level domains (radiologic recommendation, pathologic recommendation, therapy recommendation, and overall usefulness). For these paired comparisons, the effect size was approximated by Cohen *d* calculated from the distribution of paired differences. Between-phase comparisons (retrograde vs anterograde) of model ratings within each LRM were performed using Mann-Whitney *U* tests, treating cases in the 2 phases as independent samples. The same test was used to compare MDT grades between phases.

To explore the relationship between MDT quality and model performance, Spearman rank correlation coefficients (ρ) were computed between case-level MDT grade and, separately, each model’s average recommendation score and usefulness score. Additional analyses examined the correlation between MDT grade and the case-level mean of the 2 models’ average recommendation scores. Finally, MDT grades were dichotomized into 4 or less versus more than 4, and Mann-Whitney *U* tests were used to compare the distribution of average recommendation scores between these lower- and higher-quality MDT strata for each model and phase. All hypothesis tests were 2-sided. A *P* value <.05 was considered statistically significant; given the exploratory nature and the number of related comparisons, *P* values are interpreted with caution, and emphasis is placed on the magnitude and direction of effects alongside statistical significance.

Interrater reliability was assessed using the preconsensus independent scores of the 2 graders. Quadratic-weighted Cohen κ and intraclass correlation coefficients (ICC; 2,1), 2-way random, single measures, absolute agreement) were calculated for each grading domain. Given the anticipated ceiling effects in model ratings, percentage exact agreement and percentage agreement within one point were also reported as complementary reliability measures. Data preprocessing and statistical analyses were performed using the Python programming language (version 3.11), with the *pandas* library for data handling and *scipy.stats* for nonparametric tests and correlation analyses. Plots and summary tables for visualization were prepared using Python with “matplotlib” where needed.

### Ethical Considerations

The study protocol was approved by the Ethics Committee of the University Hospital of Split (approval number: 2181‐147). All procedures complied with relevant data protection regulations and General Data Protection Regulation requirements. Because this study used only deidentified, routinely collected clinical documentation and involved no direct contact with patients or interventions, individual informed consent was waived by the ethics committee. Regarding data safety in the context of LRM use: all patient reports were fully deidentified (names, dates of birth, and other direct identifiers were removed) prior to submission to the models. Both models were accessed exclusively via their commercial API end points. Under the applicable API terms of service at the time of the study, neither OpenAI nor Deepseek use data submitted through their APIs for model training or improvement purposes, thereby ensuring that deidentified patient data were not incorporated into future model weights. No data were submitted through consumer-facing chat interfaces, which may have different data-use policies.

## Results

### Overview of Cases and Ratings

The dataset comprised 100 unique real-world lung oncology cases presented at an MDT meeting, of which 50 were evaluated retrospectively (retrograde phase) and 50 prospectively while MDT members were aware of the evaluation (anterograde phase). Patient ages ranged from 45 to 87 years; 60 patients were male and 40 were female. The most common histological subtype was adenocarcinoma (47 cases), followed by squamous cell carcinoma (n=26, 26%), small cell lung carcinoma (n=16, 16%), and other or not otherwise specified subtypes (n=11, 11%). Among cases with established staging, 15 (15%) were classified as stage I, 7 (7%) as stage II-III, and 40 (40%) as stage IV; the remaining 38 (38%) cases were in the prestaging workup phase at the time of MDT discussion. Among patients with documented performance status, 37 out of 43 (86%) had an ECOG score of 0 and 6 (14%) had an ECOG score of 1. Molecular marker testing was performed in a substantial proportion of cases: PD-L1 expression was assessed in 65 (65%) cases, EGFR mutation testing in 41 (41%), and ALK rearrangement testing in 9 (9%). The distribution of histological subtypes, stages, and baseline characteristics was broadly comparable between the retrograde and anterograde phases.

**Figure 1.**
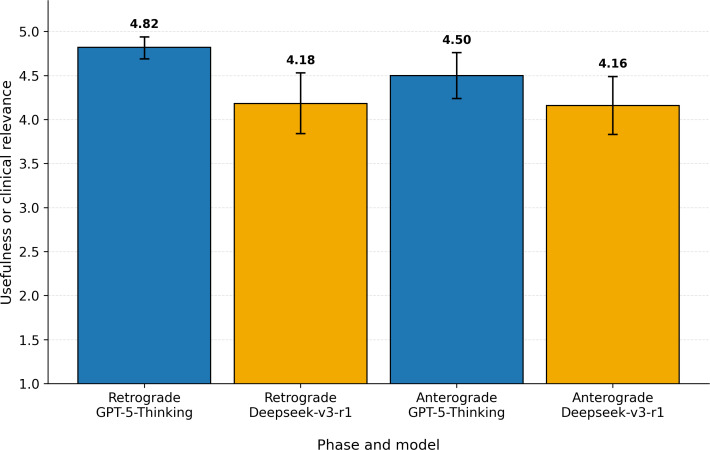
Overall usefulness or clinical relevance ratings (1‐5 Likert scale) for GPT-5-Thinking and Deepseek-v3-r1 across the retrograde and anterograde phases. Bars represent means, and error bars indicate 95% CIs*.*

Across models and phases, ratings were high with marked ceiling effects. In the retrograde phase (N=50), GPT-5-Thinking achieved mean scores of 4.89 (95% CI 4.78‐4.99) for radiologic diagnostics, 4.88 (95% CI 4.78‐4.97) for pathologic diagnostics, 4.82 (95% CI 4.69‐4.94) for oncological therapy, and 4.82 (95% CI 4.69‐4.94) for overall usefulness (Figure 1). Deepseek-v3-r1 in the same phase achieved mean scores of 4.76 (95% CI 4.62‐4.89), 4.73 (95% CI 4.57‐4.90), 4.18 (95% CI 3.82‐4.54), and 4.18 (95% CI 3.84‐4.53), respectively. In the anterograde phase (n=50), GPT-5-Thinking maintained high ratings with means of 4.94 (95% CI 4.85‐5.03) for radiologic diagnostics, 4.96 (95% CI 4.90‐5.02) for pathologic diagnostics, 4.46 (95% CI 4.18‐4.74) for oncological therapy, and 4.50 (95% CI 4.24‐4.76) for overall usefulness (Figure 1), while Deepseek-v3-r1 achieved mean scores of 4.64 (95% CI 4.47‐4.81), 4.78 (95% CI 4.65‐4.91), 4.20 (95% CI 3.86‐4.54), and 4.16 (95% CI 3.83‐4.49), respectively.

**Figure 2.**
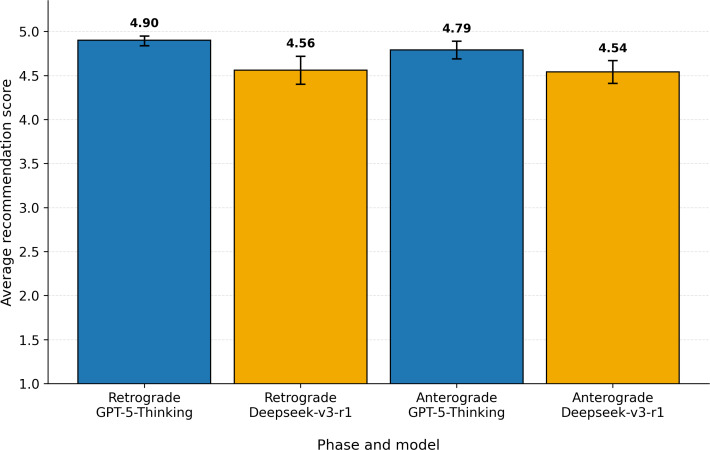
Average recommendation score (mean of radiologic diagnostics, pathologic diagnostics, and oncological therapy recommendation grades; 1‐5 Likert scale) for GPT-5-Thinking and Deepseek-v3-r1 across the retrograde and anterograde phases. Bars represent means, and error bars indicate 95% CIs.

For each model and case, an aggregate recommendation score (average recommendation score) was computed as the mean of the three recommendation-domain grades (radiologic, pathologic, and therapy). The mean average recommendation score in the retrograde phase, it was 4.90 (95% CI 4.84‐4.95) for GPT-5-Thinking and 4.56 (95% CI 4.40‐4.72) for Deepseek-v3-r1, and in the anterograde phase 4.79 (95% CI 4.69‐4.89) for GPT-5-Thinking and 4.54 (95% CI 4.41‐4.67) for Deepseek-v3-r1 (Figure 2). Case-level MDT grades were obtained by averaging the MDT grade recorded by the 2 graders for the same case and phase. The mean MDT grade was 4.14 (95% CI 3.96‐4.33) in the retrograde phase and 4.34 (95% CI 4.16‐4.52) in the anterograde phase, with no statistically significant difference between phases (Mann-Whitney *U*=1033.0, *P*=.13).

### Interrater Reliability

Preconsensus interrater reliability was assessed using the independent scores of both graders for GPT-5-Thinking outputs across all 100 cases. For model recommendation domains (radiology, pathology, therapy, and usefulness), quadratic-weighted Cohen κ and ICC(2,1) values were low (κ= −0.06 to 0.19; ICC range −0.06 to 0.19), reflecting the severe ceiling effects inherent in these ratings rather than true disagreement. Exact agreement ranged from 51.5% (pathology) to 77.2% (therapy and usefulness), and agreement within 1 Likert point was high across all domains: 99.0% for radiology, 95.0% for pathology, 91.1% for therapy, and 93.1% for usefulness. For MDT grade, which exhibited greater score dispersion, weighted κ was 0.17 and ICC(2,1) was 0.17, with 30.7% exact agreement and 69.3% agreement within one point; the 2 graders showed a systematic difference in MDT grading stringency (mean 4.26 95% CI (4.11-4.40) vs 3.30 95% CI (3.23-4.15)), which was resolved through the consensus adjudication process. These findings indicate that while traditional reliability coefficients are attenuated by ceiling effects in model ratings, the 2 graders showed substantive concordance, with the vast majority of independent scores falling within one point of each other before consensus discussion.

### Case-Level Comparison of GPT-5-Thinking and Deepseek-V3-R1

Paired analyses were conducted per case and phase, comparing GPT-5-Thinking and Deepseek-v3-r1 using Wilcoxon signed-rank tests for each domain. In the retrograde phase (50 paired cases), GPT-5-Thinking outperformed Deepseek-v3-r1 on radiologic recommendations (mean paired difference +0.24; *P*<.001), oncological therapy recommendations (+0.63; *P*<.001), and overall usefulness (+0.63; *P*<.001). In these domains, GPT-5-Thinking was rated higher than Deepseek-v3-r1 in 11/49 (22%) cases for radiologic recommendations, 15/49 (31%) cases for therapy, and 17/49 (35%) cases for usefulness; Deepseek-v3-r1 was rarely preferred. Differences in pathologic recommendations numerically favored GPT-5-Thinking (mean paired difference +0.14), but did not reach statistical significance (*P*=.15).

In the anterograde phase (50 paired cases), GPT-5-Thinking remained superior for diagnostic recommendations. Radiologic recommendations were rated higher for GPT-5-Thinking with a mean paired difference of +0.30 (*P*<.001), and GPT-5-Thinking was never rated worse than Deepseek-v3-r1 for this domain (13/50 cases favored GPT-5-Thinking; 37/50 ties). Pathologic recommendations also favored GPT-5-Thinking (mean paired difference +0.18; *P*=.008). For oncological therapy and overall usefulness, GPT-5-Thinking retained modest numerical advantages (mean paired differences +0.26 and +0.34, respectively), but these differences were not statistically significant (therapy *P*=.24; usefulness *P*=.12).

### Overall Model Grade Compared With MDT Grade

**Figure 3.**
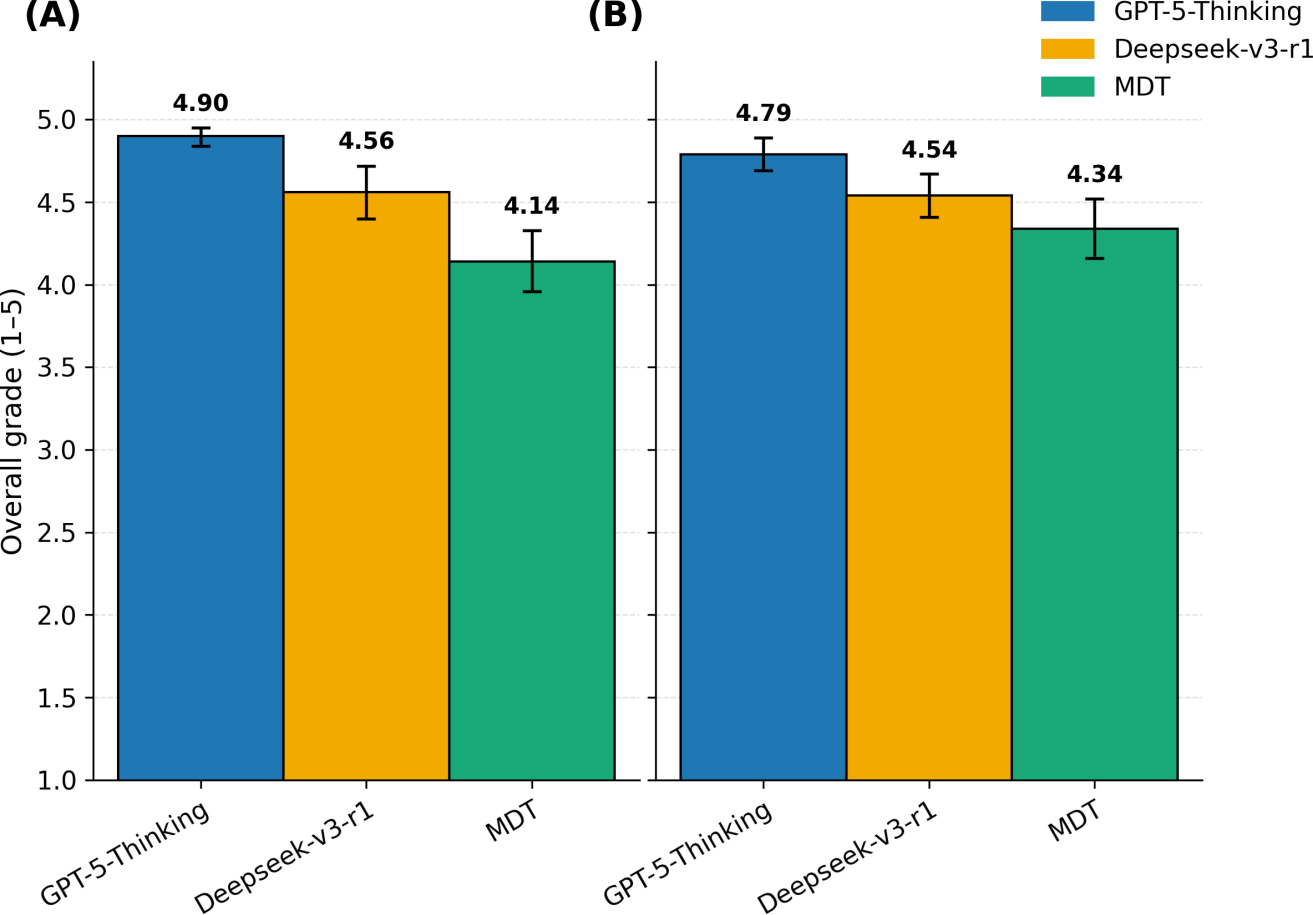
Overall grade comparison between model outputs and the human multidisciplinary team (MDT) decision, stratified by phase. (A) Retrograde phase (50 retrospective cases) and (B) anterograde phase (50 prospective cases). The overall model grade is defined as the average recommendation score (mean of radiologic, pathologic, and therapy recommendation grades; 1-5 scale), while the MDT grade reflects the expert-rated quality of the real-world MDT decision (1-5 scale). Bars show means, and error bars indicate 95% CIs.

To compare overall model recommendation quality against the human MDT decision quality, the average recommendation score was treated as an overall model grade and contrasted with the case-level MDT grade using paired Wilcoxon signed-rank tests. In the retrograde phase, GPT-5-Thinking achieved a mean overall model grade of 4.90 (95% CI 4.84-4.95) compared with a mean MDT grade of 4.14 (95% CI 3.96-4.33), corresponding to a mean paired difference of +0.76 (95% CI 0.58‐0.93; *P*<.001). Deepseek-v3-r1 also exceeded the MDT in the retrograde phase, with a mean difference of +0.41 (95% CI 0.19‐0.64; *P*<.001; Figure 3).

In the anterograde phase, GPT-5-Thinking again received higher overall grades than the MDT (mean difference +0.45, 95% CI 0.22‐0.67; *P* <.001). Deepseek-v3-r1 showed a smaller advantage over the MDT in this phase (mean difference +0.20, 95% CI −0.05‐0.45), which did not reach statistical significance (*P*=.15; Figure 3).

Overall, both LRMs achieved high recommendation-domain ratings, with GPT-5-Thinking consistently achieving the highest scores across phases. When summarized as an overall model grade, GPT-5-Thinking significantly exceeded the MDT grade in both retrograde and anterograde phases, while Deepseek-v3-r1 exceeded the MDT grade in the retrograde phase but did not exceed it in the anterograde phase.

### Relationship Between the Quality of the MDT and Model Performance

Spearman rank correlations between the MDT grade and model scores were generally weak, consistent with the pronounced ceiling effects in model ratings. In the retrograde phase, the correlation between MDT grade and the GPT-5-Thinking average recommendation score was ρ=0.278 (*P*=.05), and the correlation between MDT grade and the usefulness score was ρ=0.224 (*P*=.12). For Deepseek-v3-r1 in the same phase, correlations were ρ=0.104 (*P*=.47) and ρ=0.115 (*P*=.43), respectively. In the anterograde phase, the correlations were weak and negative for both models: GPT-5-Thinking average recommendation ρ=−0.173 (*P*=.23), usefulness ρ=−0.25 (*P*=.08); Deepseek-v3-r1 average recommendation ρ=−0.287 (*P*=.04), usefulness ρ=−0.231 (*P*=.11). When the 2 models’ average recommendation scores were combined (case-level mean), the correlation with MDT grade remained nonsignificant in the retrograde phase (ρ=0.107, *P*=.46) but reached significance in the anterograde phase (ρ=−0.380, *P*=.006), suggesting a weak inverse relationship in that cohort.

### Error Analysis of Low-Scoring Cases

To move beyond global quality scores, a post hoc error analysis was performed on all cases receiving a therapy or usefulness score ≤2 (indicating potentially inadequate or unsafe recommendations). GPT-5-Thinking had no such cases in the retrograde phase and 3 in the anterograde phase (3/50, 6%), whereas Deepseek-v3-r1 had 9 in the retrograde phase (9/50, 18%) and 7 in the anterograde phase (7/50, 14%). Review of the corresponding model outputs identified the following recurrent error patterns.

First, staging ambiguity leading to therapy overescalation was observed in multiple cases: when tumor, node, and metastasis staging was incomplete or borderline (eg, suspected but unconfirmed nodal involvement), models tended to recommend more aggressive regimens (such as concurrent chemoradiation or perioperative chemo-immunotherapy) without first recommending confirmatory staging procedures (eg, endobronchial ultrasound–guided transbronchial needle aspiration), resulting in potential overtreatment of early-stage disease. Second, safety and contraindication oversight was identified in the most critically scored cases: in one instance, immunotherapy (nivolumab) was recommended for a patient with a known aortic aneurysm and brachiocephalic thrombus without discussion of the associated vascular risk; in another, perioperative pembrolizumab was recommended without addressing the patient’s concurrent anticoagulation for atrial fibrillation. Third, inadequate integration of molecular context was noted: models sometimes failed to sufficiently weigh low PD-L1 expression (≤5%) when selecting immunotherapy-containing regimens, or did not discuss alternative chemotherapy-first strategies for PD-L1-low tumors. Fourth, in one case involving a high-grade neuroendocrine tumor (Ki-67 approximately 75%), the model recommended a regimen more appropriate for indolent well-differentiated neuroendocrine tumors rather than the platinum-etoposide backbone indicated by the aggressive tumor biology. These error patterns were substantially more frequent in Deepseek-v3-r1 outputs than in GPT-5-Thinking outputs, consistent with the global score differences reported above.

## Discussion

In this real-world evaluation of 100 consecutively discussed lung cancer cases, both LRMs produced diagnostic and therapeutic recommendations that were rated as high quality by blinded expert graders when benchmarked against the consensus output of a human MDT. GPT-5-Thinking consistently achieved the highest ratings across all assessed domains in both study phases, with a modest but reproducible advantage over Deepseek-v3-r1, particularly in radiologic and therapy recommendation domains. Both models met or exceeded expert-graded MDT decision quality, with GPT-5-Thinking significantly surpassing the MDT grade in both phases and Deepseek-v3-r1 doing so in the retrograde phase (detailed numerical results are reported in the Results section). The difference in performance between the two LRMs, despite both being state-of-the-art reasoning models, highlights that model choice matters and that comparative evaluation should be task- and context-specific, even when headline benchmarks imply parity [9]. We also found no statistically significant change in MDT recommendation quality between phases (*P*=.15), suggesting that the knowledge of being compared with AI outputs did not measurably influence the MDT’s routine decision-making; in other words, we found no strong evidence of a Hawthorne effect.

Our findings align with (and extend) recent tumor-board concordance studies, while highlighting the added value of evaluating models within real MDT workflows. In a prospective study of 100 tumor-board cases, Doğan et al [10] reported 76.4% agreement between ChatGPT-4 recommendations and MDT decisions (κ≈0.76), indicating substantial concordance even with earlier-generation GPT-4 systems. Similarly, Zabaleta et al [11] reported approximately 76% concordance (κ=0.59) between GPT-3.5 outputs and a thoracic tumor board when the model was provided detailed case data plus guideline context. In our cohort, the near-ceiling expert ratings (particularly for GPT-5-Thinking) are consistent with a high level of clinical acceptability and imply concordance at least comparable to these prior tumor-board evaluations, while also suggesting incremental gains in completeness and reliability with newer models.

At the same time, prior work suggests that model generation matters: earlier deployments of GPT-3.5 in tumor-board style tasks showed more modest concordance, such as approximately 50% agreement in breast cancer tumor board decision-making (with improvement in invasive disease subsets) [12]. Comparative tumor-board analyses in head and neck cancer similarly suggested more comprehensive and accurate recommendations from GPT-4 than GPT-3.5, mirroring the “newer-is-better” pattern we observed when comparing GPT-5-Thinking against Deepseek-v3-r1 across multiple domains [13]. These comparisons collectively support a rapid performance trajectory from early chat models toward newer LRMs that more consistently deliver guideline-aligned, workflow-compatible outputs.

Beyond tumor boards, our results also fit within the broader literature showing that LLMs can approach expert performance on oncology decision tasks and clinical reasoning benchmarks [14]. For example, Ah-Thiane et al [15] reported 72.4% concordance between GPT-4 suggestions and expert MDT recommendations across oncology scenarios. However, benchmark parity does not always translate to real workflow performance: Sandmann et al [9] found DeepSeek-v3.1 and GPT-4o to be closely matched across standardized clinical scenarios, whereas in our live-MDT-derived cases, GPT-5-Thinking showed clearer advantages over DeepSeek-v3-r1, particularly in therapy and usefulness during the retrospective phase. This discrepancy underscores the importance of evaluating LRMs not only on vignette-style benchmarks but also in “messier” real-world settings where documentation is incomplete, sequencing matters (eg, staging prerequisites), and recommendations must be operationalizable within local pathways [16].

A distinguishing feature of our study is that the clinical data provided to the models consisted of actual deidentified MDT documentation rather than curated case vignettes. Most prior studies evaluating LLMs in oncology have relied on well-structured clinical scenarios—either purpose-written vignettes, guideline-derived question banks, or retrospectively curated case summaries in which information is preorganized, complete, and internally consistent [7,16]. In contrast, the reports used in our study retained the heterogeneous, unstructured character of real electronic health records: verbatim radiology and pathology reports of varying length and detail, free-text clinical histories, laboratory values in inconsistent formats, and procedural notes from multiple providers. In some cases, staging information was incomplete (38% of cases were in the prestaging phase), findings were presented nonchronologically, or relevant data points had to be inferred from context rather than stated explicitly. This distinction is important for 2 reasons. First, it means the models were tested on a task that more closely resembles actual clinical deployment, where the ability to extract relevant information from noisy, unstructured input is itself a critical capability. Second, it suggests that the high performance observed—particularly for GPT-5-Thinking—is not contingent on receiving preprocessed, idealized inputs, which strengthens the case for the practical utility of LRMs in real MDT workflows. A representative anonymized case report is provided in Multimedia Appendix 1 to illustrate the nature and variability of the input data.

The observation that GPT-5-Thinking exceeded the expert-graded quality of MDT outputs should be interpreted carefully. MDTs remain the clinical standard for complex cancer care, and our end point was expert-rated recommendation quality rather than patient outcomes. Nevertheless, the finding is important because it highlights where AI may offer incremental value even in mature MDT environments. Unlike human teams, LRMs do not experience time pressure or intrateam variability in the same way, and they can produce a “full checklist” style response that may score highly on structured Likert criteria. At the same time, the lack of a measurable Hawthorne-type effect between retrograde and anterograde phases implies that MDT members likely relied on established practice patterns and that simple awareness of AI comparison is insufficient to materially shift decision quality in routine care. A significant number of patients globally are underserved from the MDT point of view; they are managed in small, regional, or private units, or they are living in underserved medical or social systems. For these patients and MDT members, the AI help could be more beneficial than we have measured in our study.

On the other hand, the present findings do not negate well-described risks. Prior studies have documented reasoning errors, hallucinations, and potentially unsafe recommendations under certain conditions [17,18]. Our post hoc error analysis of low-scoring cases provides concrete examples of such risks: staging ambiguity leading to therapy overescalation, failure to flag critical contraindications (eg, immunotherapy in a patient with an aortic aneurysm), insufficient weighting of molecular markers such as low PD-L1 expression, and misclassification of tumor biology in neuroendocrine tumors. These errors, though infrequent in absolute terms (especially for GPT-5-Thinking), underscore that even high-performing models can produce clinically unsafe recommendations when confronted with incomplete staging, complex comorbidity profiles, or atypical tumor biology. The difference in performance between GPT-5-Thinking and Deepseek-v3-r1 in our diagnostic domains also argues against treating “LLMs” as interchangeable and suggests that comparative evaluation should be task- and context-specific, even when headline benchmarks imply parity [9]. From a practical perspective, these models may be most useful as structured second opinions (or medical error checkers) and as preparation aids for MDT meetings. In centers with limited subspecialty coverage, they could help standardize recommendations and reduce variability, while in high-volume settings, they could assist with pre-MDT summarization, flag missing data, and propose guideline-concordant pathways that clinicians can confirm, refine, or reject. They may also have a role in internal quality assurance by systematically comparing MDT outputs to externalized best-practice patterns, and in education by providing trainees with a consistent reference baseline. However, it must be emphasized that our study evaluated LRM and MDT outputs in parallel rather than testing a collaborative human–AI workflow; therefore, we cannot conclude from these data alone that integrating LRM recommendations into MDT meetings would improve final clinical decisions. Our findings are consistent with other recent single-center comparisons showing that LLMs can approximate or exceed expert panel recommendations in structured oncology tasks [17,18], but generalizability across institutions, tumor types, and clinical workflows remains unestablished. Importantly, several studies have cautioned that high stand-alone LLM performance does not automatically translate to improved outcomes when models are used as decision aids, because of factors such as automation bias, overreliance, and disruption of established team dynamics [10]. Our error analysis, which identified clinically unsafe recommendations in a minority of cases, supports the view that any clinical deployment would need to incorporate safeguards, but the design and effectiveness of such safeguards were not tested in this study.

The limitations of our study include its single-center design and the modest sample size (100 cases; 50 per phase), which may limit generalizability to other institutions and patient populations. The Likert-based grading approach, although standardized, is inherently subjective and exhibits ceiling effects that may reduce sensitivity for detecting smaller but clinically relevant differences between systems. In addition, the LRMs function as black-box models, which restricts our ability to explain why specific recommendations were produced or to systematically anticipate failure patterns. It should also be noted that oncology is a domain with extensive publicly available clinical guidelines (eg, National Comprehensive Cancer Network and European Society for Medical Oncology), published trial data, and open-access medical literature, all of which are likely well-represented in LRM training corpora. The strong performance observed here may therefore partly reflect this training data advantage and may not generalize to clinical domains where high-quality open-access training data are more limited. Our prompting strategy, while standardized and reproducible, represents only one of many possible approaches; alternative techniques such as multiturn prompting, retrieval-augmented generation with institutional guidelines, or model-specific prompt optimization might yield different results, and our findings should be interpreted within this context (Multimedia Appendix 1), for a detailed discussion of prompt engineering considerations). Additionally, our study evaluated LRM and MDT performance independently rather than assessing the combined performance of human clinicians augmented by LRM outputs. While human–AI collaborative decision-making is arguably the most clinically relevant scenario, evaluating it retrospectively is methodologically problematic: representing the same cases to the MDT with AI recommendations would introduce recall bias and familiarity effects that would confound any comparison, and the anterograde cases were already discussed under real clinical time constraints that cannot be recreated. Our findings are also specific to the two models tested (GPT-5-Thinking and Deepseek-v3-r1), which were the state-of-the-art proprietary and open-weight LRMs at the time of data collection. Given the rapid pace of model development, successor models are likely to achieve equal or superior performance; however, the generalizability of our results to other current or future LRMs should not be assumed without empirical validation. Finally, our benchmark was expert-graded recommendation quality rather than downstream outcomes; therefore, we cannot conclude that higher-rated recommendations would translate into improved survival and more efficient patient care.

Future work should move beyond global quality scores toward more granular evaluation, including explicit identification of hallucinations, unsafe recommendations, and omission errors. Improving interpretability—whether through structured rationales, retrieval-based citation of guideline sources, or standardized explanation templates—would likely be important for clinician trust and medico-legal defensibility. It would also be valuable to assess the impact of local adaptation (eg, fine-tuning or retrieval augmentation using institutional pathways and national guidelines) on performance and acceptance. Critically, future prospective studies should evaluate human–AI collaborative decision-making, in which MDT members receive LRM-generated recommendations before or during case discussions, and the resulting hybrid decisions are compared to the performance of unassisted MDTs. Such studies would clarify whether LRM outputs genuinely improve final decision quality, alter team dynamics, or introduce new sources of automation bias. Finally, prospective studies should also examine workflow outcomes, such as time-to-decision, completeness of pre-MDT data, and clinician cognitive load.

In conclusion, in a real-world cohort of consecutive lung cancer cases at a single center, advanced LRMs (particularly GPT-5-Thinking) generated diagnostic and therapeutic recommendations that were rated as higher quality than expert-graded MDT decisions. While these findings suggest potential for LRMs as clinical decision support tools, the single-center design, ceiling effects in grading, and absence of a prospective human–AI collaborative evaluation limit the conclusions that can be drawn about real-world clinical integration. Prospective multicenter studies evaluating the impact of LRM-assisted MDT workflows on decision quality and patient outcomes for patients with lung cancer are needed before implementation recommendations can be made.

## Supplementary material

10.2196/91733 Multimedia Appendix 1 Representative anonymized case report, full prompt template, corresponding GPT-5-Thinking output, and prompt-engineering details.

## Abbreviations

LLM: large language model
LRM: large reasoning model
MDT: multidisciplinary team
