# Supplementary material for: Evaluating Large Reasoning Models Versus Human Multidisciplinary Teams in Lung Cancer Decision-Making: Real-World Study
Source: J Med Internet Res. 2026 Jul 16;28:e91733. doi: 10.2196/91733 (PMC13374795; doi:10.2196/91733)
Supplement: Multimedia Appendix 1 [file jmir-v28-e91733-s001.docx]

# **Supplementary Material**

*Evaluating Large Reasoning Models Versus Human Multidisciplinary Teams in Lung Cancer Decision-Making: A Real-World Study*

# Appendix A. Standardized Prompt Template Used for LRM Querying

The following prompt was used identically for both GPT-5-Thinking and Deepseek-v3-r1 across all 100 cases. The [PATIENT REPORT] placeholder was replaced with the de-identified MDT case summary for each individual case. No prompt modifications were made between cases or between models.

**Persona**

You are to assume the role of a world-class, board-certified Medical Oncologist with over 20 years of experience participating in Multidisciplinary Team (MDT) meetings at a leading cancer center. You are an expert in the latest clinical guidelines (including NCCN and ESMO), cutting-edge clinical trials, and personalized cancer therapy. Your primary strength is synthesizing complex patient information into clear, actionable recommendations for a team of specialists.

**Primary Objective**

Your task is to provide expert decision support for an oncology MDT meeting. You will be given a patient’s medical report. Based only on the information in this report and your extensive expert knowledge, you will generate a comprehensive analysis and set of recommendations. Your output will be used by oncologists, surgeons, radiologists, pathologists, and other members of the MDT to facilitate a collaborative and evidence-based treatment plan.

**Instructions for Analysis**

**1. Careful Scrutiny:** Meticulously analyze the entire provided patient report. Extract all relevant details, including patient demographics, medical history, comorbidities, chronic medications, diagnosis, cancer stage (TNM), histology, molecular and biomarker results (e.g., PD-L1, MSI, specific mutations), and previous treatments.

**2. Identify Gaps:** If critical information for decision-making is missing from the report, explicitly state what is missing and why it is important.

**3. Evidence-Based Approach:** Ground all your recommendations in the most current, internationally recognized clinical guidelines (e.g., NCCN, ESMO). If relevant, reference key clinical trials that support your proposed therapeutic regimen.

**4. Patient-Centricity:** Always consider the patient’s overall clinical context, including age, performance status, and known comorbidities mentioned in the report, when formulating recommendations.

**Required Output Format**

The prompt specified a structured markdown output format comprising four sections:

**1. Case Summary** – Patient identifiers, full diagnosis (histology, grade, stage), and key report findings.

**2. Recommendations for Further Diagnostics** – Additional tests, imaging, or consultations, each with a brief rationale.

**3. Therapeutic Recommendations** – Primary recommendation (exact regimen, rationale citing guidelines, expected goals) and alternative/second-line options.

**4. Key Discussion Points for MDT** – Areas of uncertainty, potential challenges, or critical decision points for team deliberation.

# Appendix B. Prompt Engineering Rationale

The prompt was designed following established principles of prompt engineering for clinical applications. Several deliberate design choices were made:

**Role assignment (persona prompting):** The prompt assigned the model the role of a board-certified medical oncologist with extensive MDT experience. This technique, sometimes referred to as “persona prompting,” has been shown to improve domain-specific performance in large language models by anchoring the model’s outputs within a relevant expert framework and reducing the likelihood of generic or non-specialist responses.

**Structured output specification:** The prompt explicitly defined the required output sections (case summary, diagnostic recommendations, therapeutic recommendations, and discussion points). This structured approach ensured that model outputs were directly comparable across cases and between models, and that all clinically relevant domains were systematically addressed. Without such constraints, model outputs may vary in scope and organization, complicating expert grading.

**Evidence grounding:** The prompt explicitly instructed the models to cite current clinical guidelines (NCCN, ESMO) and relevant clinical trials. This design choice was intended to promote guideline-concordant outputs and to provide graders with transparent reasoning for the recommendations.

**Single-turn, zero-shot design:** Each case was submitted as a single prompt without iterative refinement or few-shot examples. This zero-shot approach was chosen to reflect a realistic deployment scenario in which clinicians would submit a case and receive a recommendation without engaging in multi-turn dialogue. While iterative prompting or chain-of-thought elicitation might have improved outputs in specific cases, the single-turn design ensures reproducibility and avoids post-hoc selection bias.

**Identical prompting across models:** The same prompt template was used for both GPT-5-Thinking and Deepseek-v3-r1 without model-specific optimization. This approach ensures a fair comparison but may underestimate the performance of either model under individually optimized conditions.

It is important to acknowledge that prompting strategy can significantly influence LRM outputs. Alternative approaches—such as multi-turn prompting, retrieval-augmented generation (RAG) with institutional guidelines, few-shot examples with exemplar MDT cases, or model-specific prompt tuning—might yield different results. Our findings therefore reflect the performance of these models under a standardized, zero-shot, single-prompt paradigm and should not be interpreted as an upper bound on LRM capability for clinical decision support. Future studies should systematically evaluate the impact of different prompting strategies on clinical recommendation quality.

# Appendix C. Representative Anonymized Case Report

The following is a representative example of a de-identified case report as it was provided to the LRMs. This example illustrates the unstructured, heterogeneous character of real-world MDT documentation. All identifying information has been replaced with fictitious data; clinical details have been modified to prevent re-identification while preserving the structural characteristics of the original reports.

**Patient X.Y.**

**Anamnesis (from request):**

Adenocarcinoma pulmonum pT1bN0

Lobectomy of the left upper lung lobe via VATS-uniport, Mediastinal lymphadenectomy (groups 5, 6)

Emphysema, Bronchiectasis

Hypertension, DVT of the right leg

ECOG 0.

**Anamnesis:**

No complaints. Functions normal. Former smoker, quit 6 months ago. 1x48 PY. Does not consume alcohol. Allergies: none manifested.

**PET/CT (low-dose CT):**

Very intense uptake (SUVmax=25.4) is seen in a nodular change in the upper left lung lobe. Elsewhere in the lung parenchyma, hilum, and mediastinum, no increased uptake. Liver homogeneously displayed, without focal pathological uptake. In the displayed parts of the skeleton, no focal pathological uptake. Increased FDG uptake (SUVmax=4.5) in a small nodular change in the left parotid. Conclusion: pathological FDG metabolism in the nodular change of the upper left lung lobe is of malignant character. Small nodular change in the left parotid – differential diagnosis Warthin’s tumor.

**MSCT:**

Inhomogeneous nodule 15mm in the left upper lobe. Diffuse emphysematous changes. Axillary, hilar, and mediastinal areas show no pathologically enlarged lymph nodes. No pleural effusion. Liver, pancreas, and spleen of appropriate size without focal lesions. Kidneys appropriate without lithiasis. Skeleton without pathological remodeling.

**Pathology report:**

SAMPLE: Upper left lobe of the left lung; Lymph nodes group 5 and 6. TUMOR SIZE: 1.7x1.6x1.5 cm. TUMOR FOCALITY: single tumor. HISTOLOGICAL TYPE: invasive non-mucinous adenocarcinoma. ARCHITECTURAL SUBTYPE: solid (70%), papillary (20%), tubular (10%). HISTOLOGICAL GRADE: G3. VISCERAL PLEURA INVASION: not found. RESECTION MARGINS: not involved by tumor. LYMPH NODES: 0/4 positive.

**Molecular testing:**

EGFR: no mutation detected. ALK IHC: negative. PD-L1 (22C3): TPS 40%.

***Note:*** *This example illustrates a relatively well-documented case. In practice, case reports varied considerably: some lacked molecular testing results, others presented findings non-chronologically, and a substantial proportion (38%) were in the pre-staging workup phase with incomplete staging information at the time of MDT discussion.*

# Appendix D. Representative GPT-5-Thinking Model Output

The following is the GPT-5-Thinking output generated for the anonymized case presented in Appendix C. The output has been lightly formatted for readability without altering clinical content. This example illustrates the structured format of model responses, including case summary, diagnostic recommendations, therapeutic recommendations with rationale, and key MDT discussion points.

**1. Case Summary:**

**Patient:** X.Y., age not stated (male, ECOG 0)

**Diagnosis:** Invasive non-mucinous lung adenocarcinoma, solid-predominant (70%), papillary (20%), tubular (10%); grade 3; pT1bN0M0 (stage IA2, 1.7 cm); R0 after left upper lobectomy (VATS)

**Key Report Findings:** PD-L1 (22C3) TPS 40%; EGFR wild-type; ALK IHC negative; Former heavy smoker (48 PY, quit 6 months ago); Comorbidities: emphysema, bronchiectasis, hypertension, history of DVT right leg; Post-operative imaging shows no residual disease

**2. Recommendations for Further Diagnostics:**

• Consider expanded molecular profiling (NGS panel) on tumor tissue to identify additional targetable mutations (e.g., KRAS G12C, BRAF V600E, MET exon 14, RET, ROS1, NTRK, HER2) that may influence surveillance strategy or future recurrence management. Rationale: EGFR and ALK are negative, but additional actionable targets may exist given adenocarcinoma histology.

• Baseline pulmonary function testing if not already performed, given underlying emphysema and bronchiectasis, to quantify respiratory reserve and guide any future adjuvant therapy tolerability assessment.

• Follow-up of parotid incidentaloma (SUVmax 4.5 on PET/CT, differential diagnosis Warthin tumor): consider ultrasound-guided fine-needle aspiration if interval growth or clinical concern.

**3. Therapeutic Recommendations:**

**Primary Recommendation: Adjuvant atezolizumab (immunotherapy) followed by active surveillance.** Regimen: Atezolizumab 1200 mg IV q3w for up to 16 cycles (1 year), per IMpower010 protocol. Rationale: Stage IB-IIIA resected NSCLC with PD-L1 TPS ≥1% is eligible for adjuvant atezolizumab per NCCN guidelines (category 2A for stage II-IIIA, category 2B for high-risk IB). Although pT1bN0 (stage IA2) falls below the standard threshold, high-risk features (G3, solid-predominant, 70%) combined with PD-L1 TPS 40% provide a strong biological rationale. The IMpower010 trial demonstrated significant DFS benefit in the PD-L1 ≥1% population, with the strongest benefit observed at higher PD-L1 levels. Expected goals: reduce recurrence risk; curative intent.

**Alternative: Active surveillance alone (observation).** Schedule: Contrast-enhanced CT chest every 6 months for 2 years, then annually to year 5 (NCCN v4.2024). Considerations: Stage IA2 is technically below the approved indication for adjuvant immunotherapy and adjuvant chemotherapy; no level-1 evidence supports adjuvant platinum doublet in tumors <4 cm. Surveillance alone is reasonable, particularly if the patient or MDT determines that the risks of immunotherapy (pneumonitis in a patient with underlying emphysema, potential DVT exacerbation) outweigh the uncertain benefit in this borderline indication.

**4. Key Discussion Points for MDT:**

• Do the high-risk histologic features (G3, solid-predominant, 70%) and high PD-L1 (40%) justify adjuvant immunotherapy in a formally stage IA2 patient? This represents an off-label but biologically plausible use of atezolizumab.

• Risk of immune-related pneumonitis in a patient with pre-existing emphysema and bronchiectasis: pulmonology co-management recommended if adjuvant immunotherapy is pursued.

• DVT history: assess need for ongoing anticoagulation and potential interaction with immunotherapy (immune-mediated thrombotic events).

• Parotid incidentaloma management: determine whether biopsy is needed or whether interval imaging surveillance is sufficient.

• Smoking cessation reinforcement and pulmonary rehabilitation given emphysema and recent heavy smoking history.

*Note: This output has been adapted to correspond to the anonymized case in Appendix C. Clinical details were modified consistently with the de-identification applied to the input case report; the structural format and level of detail are representative of actual GPT-5-Thinking outputs across the study.*
